# Cervical cancer screening in high-altitude areas in China: A large cross-section study of 25,173 women in northern Tibet

**DOI:** 10.3389/fonc.2022.841547

**Published:** 2022-08-25

**Authors:** Qimin Wang, Yingying He, Fang Long, Chaoran Li, Zhuowei Shen, Dongxing Guo, Duoji Zhaxi, Lamu Bumu, Zhengyu Hua, Zhigang Sun, Nan Jiang, Xu Han, Jing Li, Keqing Yan, Siqi Bai, Muhan Tao, Xiaoguang Xu, Zhen Xiao

**Affiliations:** ^1^ Institute of High Altitude Medicine, People’s Hospital of Nagqu affiliated to Dalian Medical University, Nagqu, China; ^2^ Department of Pathology, Second Affiliated Hospital of Dalian Medical University, Dalian, China; ^3^ Department of Pathology, Dalian Maternal and Child Medical Group, Dalian, China; ^4^ Department of Obstetrics and Gynecology, First Affiliated Hospital of Dalian Medical University, Dalian, China; ^5^ Department of Obstetrics and Gynecology, People’s Hospital of Nagqu affiliated to Dalian Medical University, Nagqu, China; ^6^ Performance and Quality Management Department, First Affiliated Hospital of Dalian Medical University, Dalian, China; ^7^ Department of Pathology, First Affiliated Hospital of Dalian Medical University, Dalian, China; ^8^ Department of Neurosurgery, Second Affiliated Hospital of Dalian Medical University, Dalian, China

**Keywords:** cervical cancer, HPV, cervical cancer screening, genotype, disease distribution

## Abstract

**Background:**

Cervical cancer has become a worldwide concern owing to its high incidence and mortality rates. To date, high-altitude areas of Tibet have not benefited from any large-scale cervical cancer screening programs. Therefore, we initiated a screening program to investigate the prevalence of human papilloma virus (HPV) and HPV genotype distribution to reveal cervical cancer and its precursor which lead to morbidity among women in the city of Nagqu in northern Tib3et.

**Methods:**

A total of 25,173 women were recruited to undergo HPV genotype tests between June and December 2019. Women infected with HPV 16 and/or 18 underwent colposcopy and histological examination. Women with other high-risk HPV type (hr-HPV) underwent cytological tests to determine whether to conduct further colposcopy and histological examination for diagnosis. HPV prevalence was calculated in the total population and further stratified according to various parameters, such as age group, area location (altitude level), and single or mixed infection status. The HPV genotype distribution was also investigated accordingly. Cervical lesions revealed by further colposcopic findings were also analyzed; high-grade and malignant lesion morbidities were calculated in total and in each county. Most data were collected and analyzed using descriptive and consistency check statistical methods, and a risk factor investigation for HPV infection was performed using logistic regression models.

**Results:**

The total HPV infection rate among women in Nagqu was 13.42%. Of the 25,173 women in the study, 999 (3.97%) were HPV 16/18 positive, 2,379 (9.45%) were other hr-HPV-positive, and 21,795 (86.58%) were HPV-negative. The five most common HPV genotypes, accounting for more than 60% of all HPV infections in Nagqu people, were HPV 16, 58, 31, 18, and 52. Tibetan women younger than 20 years and older than 60 years were the two age groups with the highest rates of HPV infection, 26.7% and 19.8%, respectively. Among the HPV-positive women, 2,656 (78.33%) were infected with a single strain and 732 (21.67%) were infected with multiple strains (more than two genotypes). HPV prevalence increased in high-altitude areas (positive rate highest in Nyima with an altitude of 5,000 m, 23.9%) and decreased in relatively low-altitude areas (positive rate lowest in Lhari with an altitude of 4,000 m, 6.6%). Multiple analyses showed that age, parity, age at first delivery, and altitude of residence were independent factors facilitating HPV infection in Tibetan women. High-grade and malignant cervical lesions revealed by histological findings were different among living locations, with the highest rates in Xainza, Baingoin, and Nyainrong, these being 2.019%, 1.820%, and 1.116%, respectively, among women in these areas.

**Conclusion:**

Our survey provides an overall perspective on HPV genotype infection and cervical lesions in women in northern Tibet. The data not only provide useful information for the treatment of cervical lesions but also has great value in terms of the primary and secondary prevention measures that can be taken for women living in these regions.

**Clinical Trial Registration:**

www.chictr.org.cn, indentifier ChiCTR2000035061.

## 1 Introduction

Cervical cancer is the third most common cancer worldwide and the fourth most common cancer among Chinese women. Reportedly, there are 34,000 and 275,000 cervical cancer-related deaths in China and globally, respectively (1). Human papillomavirus (HPV) has been confirmed as a pathogenic cause of cervical invasive cancer and its precancerous lesions, and cervical cancer is now regarded as a preventable disease (2).

Several cervical screening programs have been developed and applied to populations in various countries to identify precursors of cervical cancer. Multiple strategies are available for cervical cancer screening. The cheapest and simplest method of visual inspection with acetic acid has a high false-positive rate, requires a large number of trained clinicians, and is time-consuming, all of which make it difficult to access in Tibet despite its relative simplicity and low cost. The pap smear or cytology test has been used for several decades (3, 4) ; however, it has low sensitivity and specificity. Primary cytology with HPV triage has been widely used in women older than 30 years (testing HPV together with cytology), and it has high sensitivity and specificity (5–7). However, these tests are expensive and require abundant medical resources. Recently, another screening method solely for HPV testing (as the primary screening test) has been developed following cytology triage (8, 9). This method is extensively used in European countries, and it has an enhanced negative predictive value (10, 11). This screening method allows cervical cancer screening safety intervals to be extended from 3 to 5 years (12).

In most parts of China, for cervical cancer screening, we adopt the principle of “Opportunistic screening.” That is, when a woman comes to the hospital for various reasons, doctors suggest additional cervical cancer screening. Usually, combined cytology and HPV tests are used in most Chinese medical affiliations, and patients can afford the expense by themselves (13, 14). However, this strategy has not yet been widely used in Tibet. This is because Tibet has a relatively small population, large territory, and limited medical resources (15). Moreover, the majority of women in Tibet rarely visit hospitals voluntarily, except in emergencies, and they are uninclined to pay for, even affordable, screening programs. Therefore, government-financed HPV primary screening programs for all the population at right ages (16) may be the best choice for women in Tibet.

This is the first large-scale cervical cancer screening program conducted in the city of Nagqu, northern Tibet. This study aimed to investigate the prevalence and distribution of HPV genotypes and cervical lesions in Nagqu women. This is the first regional epidemiological study to elucidate the incidence of cervical cancer and its precursor which leads to morbidity in Nagqu.

## 2 Materials and methods

### 2.1 Study population and screening strategy

**Figure 1:**
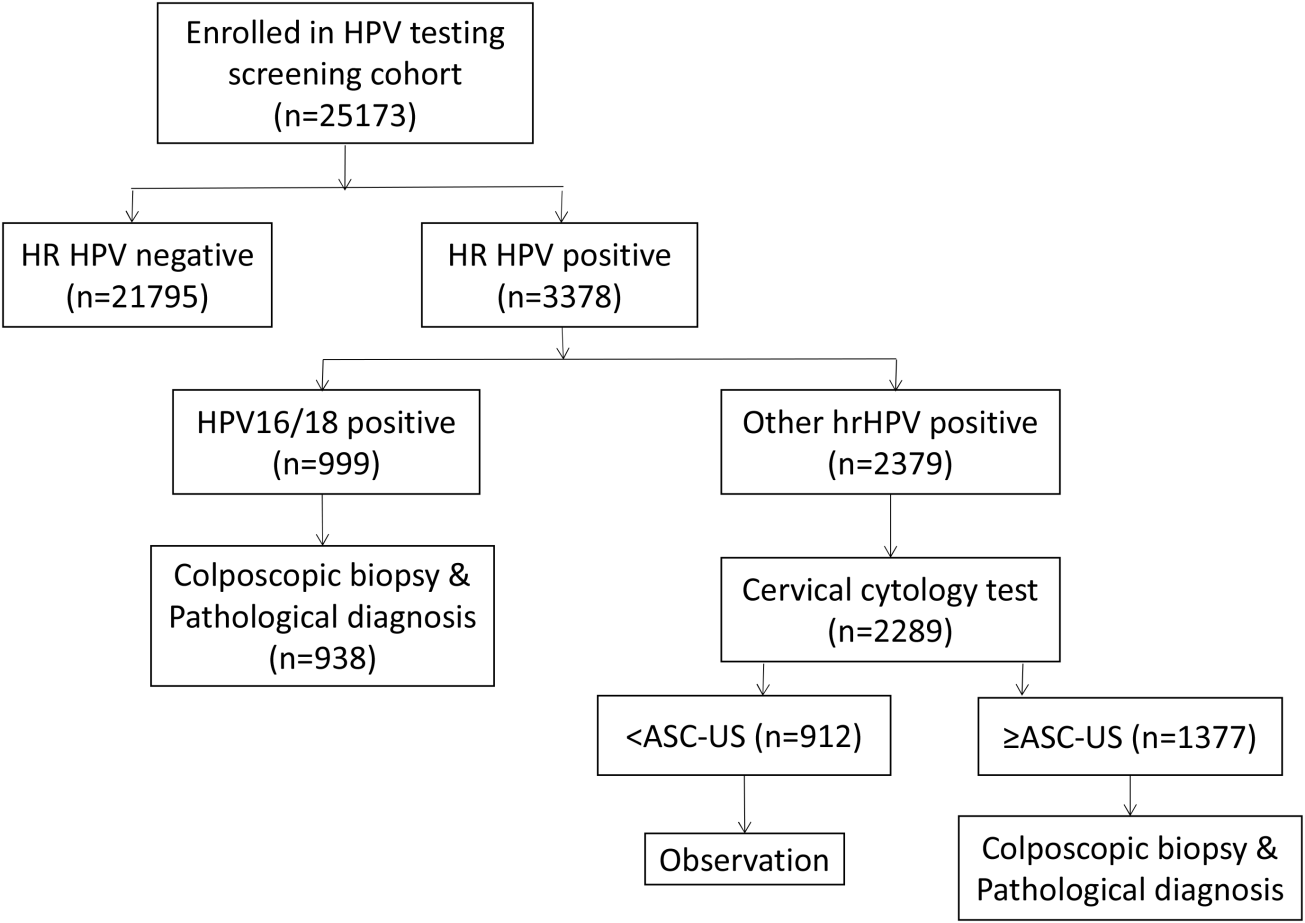
Flow of participants in the screening cohort.

A total of 25,173 married women aged 18–65 years in Nagqu City, Tibet Autonomous Region of China, who were available at home and who consented to undergo screening between June and December 2019 were recruited in this study. HPV genotype testing was the first step for all these participants. Women with positive results were divided into two groups. The first group included those with HPV 16 and/or HPV 18 infections, and the second group included high-risk HPV (hr-HPV) infections with genotypes other than 16 and 18. Women with HPV 16 and/or 18 infection in the first group underwent colposcopy and histological examination at the People’s Hospital of Nagqu. Women in the second group with other types of hr-HPV infection underwent cytology testing initially. If cytology results showed ASCUS (atypical squamous cells, undetermined significance) or more in these women, they were requested to undergo further colposcopy and histological examination for diagnosis. The flow diagram (**Figure 1**) summarizes this process.

### 2.2 HPV and cytology testing

All HPV samplings were performed by trained general practitioners at every primary hospital in all parts of Nagqu City. HPV samples were analyzed using a BGI 16-type HPV genotyping sequencing assay (BGI, Shenzhen, China). HPV testing was performed using an HPV genotyping sequencing system. This assay could detect 16 hr-HPV genotypes (16, 18, 31, 33, 35, 39, 45, 51, 52, 53, 56, 58, 59, 66, 68, and 73), as described here in detail (17, 18). Briefly, this assay aimed to target and amplify DNA of the HPV L1 gene. MiSeq or personal genome machine (PGM) sequencing strategies were used for testing. HPV16/18-positive samples were subjected to histological examination while cytology tests were also maintained for these participants for study purposes; hr-HPVs other than 16/18 infected women were referred for cytology analysis with liquid-based cytology (LBC) (Thin Prep, Hologic, Boxborough, Massachusetts, USA).

### 2.3 Colposcopy examination

Women who required colposcopy examinations were referred to a single expert gynecologist at the People’s Hospital of Nagqu, Tibet. All examinations were conducted according to routine procedures described previously (19). Hematoxylin and eosin (H&E) staining was performed for histological diagnosis.

### 2.4 Statistical analysis

SPSS 21.0 (version 21.0; SPSS, Inc. Chicago, IL, USA) was used for statistical analysis. All participants were divided by age group and county to calculate the prevalence of the overall HPV genotype, HPV 16/18, and other hr-HPV genotypes. Additionally, a stratifying strategy was used to analyze the cytology and histology results. To compare differences in HPV infection prevalence and cervical lesion rates among various locations and age groups, the chi-square test was used accordingly. Also, a logistic regression model was used to investigate factors influencing HPV infection. The p value was considered significant when p < 0.05.

## 3 Results

### 3.1 Overview of HPV infection in the women of Nagqu, north of Tibet

#### 3.1.1 Distribution of HPV genotypes in the women of Nagqu

**Figure 2:**
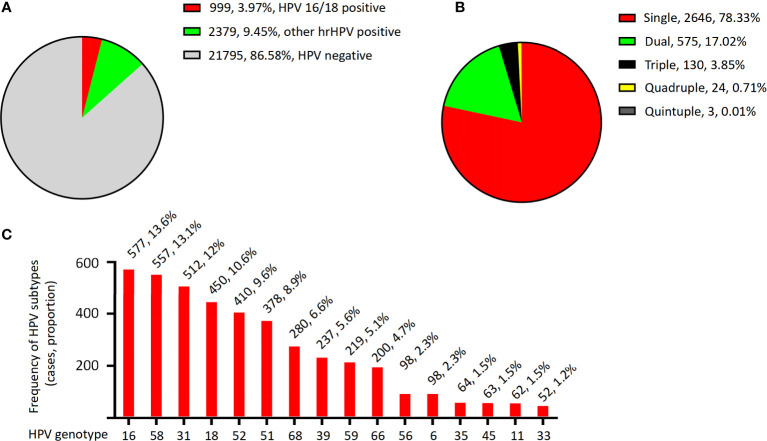
The HPV infection distribution. **(A)** Distribution of HPV genotypes in the women of Nagqu. **(B)** Distribution of multiple and simple HPV infection in the women of Nagqu. **(C)** Overall prevalence distribution of each HPV genotype.

The total HPV infection rate among women in Nagqu was 13.42%. Of the 25,173 women in the study, 999 (3.97%) were HPV 16/18 positive, 2,379 (9.45%) were other hr-HPV HPV positive, and 21,795 (86.58%) were HPV negative (**Figure 2A** and **Table 1**).

**Table 1:** Prevalence of HPV genotypes in all patients and prevalence of HPV genotypes in simple- and multiple-HPV-infection patients.

Overall infection			Simple infection			Dual infection			Triple infection			Quadruple infection			Quintuple infection		
HPV genotype	Frequency	Proportion (%)	HPV genotype	Frequency	Proportion (%)	HPV genotype	Frequency	Proportion (%)	HPV genotype	Frequency	Proportion (%)	HPV genotype	Frequency	Proportion (%)	HPV genotype	Frequency	Proportion (%)
**HPV16**	577	13.6	**HPV16**	392	14.8	**HPV31**	151	13.4	**HPV31**	48	12.1	**HPV16**	11	15.5	**HPV18**	2	13.3
**HPV58**	557	13.1	**HPV58**	364	13.8	**HPV58**	142	12.6	**HPV58**	44	11.1	**HPV18**	11	15.5	**HPV51**	2	13.3
**HPV31**	512	12	**HPV31**	302	11.4	**HPV16**	140	12.4	**HPV52**	42	10.6	**HPV31**	10	14.1	**HPV59**	2	13.3
**HPV18**	450	10.6	**HPV18**	271	10.2	**HPV18**	128	11.3	**HPV51**	41	10.3	**HPV51**	9	12.7	**HPV52**	2	13.3
**HPV52**	410	9.6	**HPV52**	245	9.3	**HPV52**	114	10.1	**HPV18**	38	9.5	**HPV52**	7	9.9	**HPV66**	2	13.3
**HPV51**	378	8.9	**HPV51**	231	8.7	**HPV51**	95	8.4	**HPV59**	35	8.8	**HPV39**	6	8.5	**HPV16**	1	6.6
**HPV68**	280	6.6	**HPV68**	181	6.8	**HPV68**	71	6.3	**HPV16**	33	8.3	**HPV58**	6	8.5	**HPV68**	1	6.6
**HPV39**	237	5.6	**HPV39**	149	5.6	**HPV59**	66	5.9	**HPV68**	27	6.8	**HPV35**	3	4.2	**HPV31**	1	6.6
**HPV59**	219	5.1	**HPV66**	127	4.8	**HPV39**	58	5.1	**HPV39**	24	6	**HPV59**	3	4.2	**HPV35**	1	6.6
**HPV66**	200	4.7	**HPV59**	113	4.3	**HPV66**	52	4.6	**HPV66**	19	4.8	**HPV45**	2	2.8	**HPV58**	1	6.6
**HPV56**	98	2.3	**HPV56**	62	2.3	**HPV6**	28	2.5	**HPV11**	13	3.3	**HPV56**	2	2.8			
**HPV6**	98	2.3	**HPV6**	62	2.3	**HPV56**	25	2.2	**HPV56**	9	2.3	**HPV11**	1	1.4			
**HPV35**	64	1.5	**HPV33**	39	1.5	**HPV35**	18	1.6	**HPV6**	8	2						
**HPV45**	63	1.5	**HPV11**	36	1.4	**HPV45**	18	1.6	**HPV35**	7	1.8						
**HPV11**	62	1.5	**HPV45**	36	1.4	**HPV11**	12	1.1	**HPV45**	7	1.8						
**HPV33**	52	1.2	**HPV35**	35	1.3	**HPV33**	10	0.9	**HPV33**	3	0.8S						

#### 3.1.2 Distribution of multiple and simple HPV infection in the women of Nagqu

For the HPV-positive patients, the genotyping results showed that 2,646 (78.33%) were singly infected, 575 (17.02%) were doubly infected, 130 (3.85%) were triple infected, 24 (0.71%) were quadruply infected, and only three patients (0.01%) were quintuply infected (**Figure 2B** and **Table 1**).

#### 3.1.3 Overall prevalence distribution of each HPV genotype

The overall prevalence of each HPV genotype in the HPV-positive patients is shown in **Figure 2C**; **Table 1**. The most frequently detected HPV genotype was HPV 16, which was detected in 577 women (13.6%). The relatively less common genotypes were HPV 58 (557, 13.1%), HPV 31 (512, 12%), HPV 18 (450, 10.6%), HPV 52 (410, 9,6%), HPV 51 (378, 8.9%), HPV 68 (280, 6.6%), HPV 39 (237, 5.6%), HPV 59 (219, 5.1%), HPV 66 (200, 4.7%), HPV 56 (98, 2.3%), HPV 6 (98, 2.3%), HPV 35 (64, 1.5%), HPV 45 (63, 1.5%), HPV 11 (62, 1.5%), and HPV 33 (52, 1.2%). In patients with single and multiple HPV infections, the HPV prevalence was slightly different from the total prevalence, as summarized in **Table 1**. For example, in single- and dual-infection groups, the four most common genotypes were HPV 16, 58, 31, and 18; however, in patients infected with three HPV genotypes, the four most common infection genotypes were HPV 31, 58, 52, and 51.

#### 3.1.4 Distribution of HPV infection in different age groups in the Nagqu area

Based on the age groups, as shown in **Table 2**, women with a younger age (less than 30 years) and older age (>50 years) had higher total HPV infection rates of 15.9% and 18.4%, respectively, *p* < 0.01. Women with a median age of (31–50 years) had a lower positive rate (12.6%), and the difference was statistically significant.

**Table 2:** Prevalence of HPV genotypes in women of different ages.

Age group(y)	Total No.	HPV16/18 infection		Other HPV infection		Mixed HPV infection		Four most frequently infected genotypes				Total	
		Positive No.	Positive, %	Positive No.	Positive, %	Positive No.	Positive, %	Top1 genotype (No., %)	Top2 genotype (No., %)	Top3 genotype (No., %)	Top4 genotype (No., %)	Positive No.	Positive, %
≤30	572	28	4.9%	56	9.8%	18	3.1%	HPV16 (22,24.2)	HPV31 (17,18.7)	HPV58 (11,12.1)	HPV52 (10,10.9)	84	14.7%
31-50	18503	694	3.8%	1635	8.8%	427	2.3%	HPV16 (403,17.3)	HPV58 (370,15.9)	HPV52 (328,14.1)	HPV18 (309,13.3)	2329	12.6%
≥51	5171	274	5.3%	679	13.1%	284	5.5%	HPV31 (163,17.1)	HPV58 (162,16.9)	HPV16 (150,15.7)	HPV18 (133,13.9)	953	18.4%

#### 3.15 Area-specific distribution of HPV infections in Nagqu

**Figure 3:**
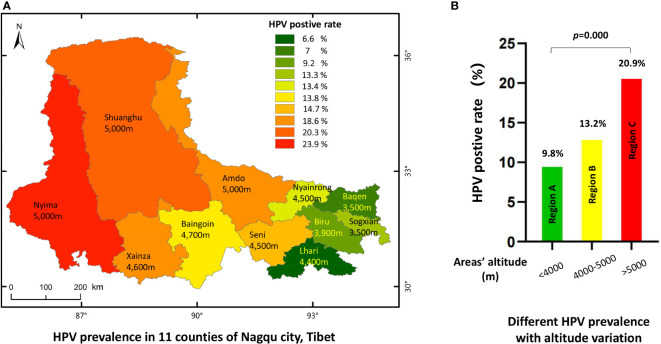
The HPV prevalence in 11 countries of Nagqu. **(A)** HPV prevalence in 11 counties of Nagqu City, Tibet. **(B)** Different HPV prevalence with altitude variation.

As the city of Nagqu has an area of 370,000 km^2^, and its 11 counties have different altitudes and different modes of production and life, we calculated the HPV infection rate in women according to each of the 11 areas. Generally, women living in eastern areas with lower altitudes had lower HPV infection rates than those living in western areas with higher altitudes (**Figure 3A** and **Table 3**). HPV infection rates in women were lowest in Lhari, Bagen, and Biru counties at 6.6%, 7%, and 9.2%, respectively. The highest infection rate was noted in the ultra-high-altitude areas of Amdo (18.6%), Nyima (23.9%), and Shuanghu (20.3%). When we combined the 11 counties into three regions according to altitude level (region A, altitude level <4,000 m; region B, altitude level within 4,000–5,000 m, region C altitude level >5000 m), we found that regions with lower altitudes had the lowest HPV-positive rate of 9.8%, while the rates in regions B and C were 13.2% and 20.9%, respectively (*p* = 0.001). Correlation analysis also indicated a significant correlation between altitude and HPV infection rates, *p* = 0.001, gamma = -0.251 (**Figure 3B**).

**Table 3:** Prevalence of HPV genotypes in women from 11 counties of Nagqu city, Tibet.

District	Average Altitude (m)	Total No.	HPV16/18 infection		Other hrHPV infection		Mixed hrHPV infection		Four most frequent infected subtypes				Total	
			Positive No.	Positive, %	Positive No.	Positive, %	Positive No.	Positive, %	Top1	Top2	Top3	Top4	Positive No.	Positive, %
Sogxian	3,500	2284	111	4.9	193	8.5	60	19.1	HPV16 (69,22.7%)	HPV18 (42,13.8%)	HPV58 (35,11.5%)	HPV31 (32,10.5%)	304	13.3
Biru	3,900	4306	107	2.5	289	6.7	71	17.9	HPV16 (71,17.9%)	HPV58 (47,11.9%)	HPV52 (44,11.1%)	HPV51 (42,10.6%)	396	9.2
Lhari	4,400	2981	45	1.5	152	5.1	37	18.8	HPV16 (29,14.7%)	HPV58 (26,13.2%)	HPV31 (22,11.2%)	HPV52 (21,10.67%)	197	6.6
Baqen	4,500	1783	38	2.1	87	4.9	21	16.8	HPV16 (44,18.2%)	HPV58 (33,13.6%)	HPV31 (32,13.2%)	HPV18 (24,9.9%)	125	7
Seni	4,500	4004	165	4.1	422	10.5	114	19.4	HPV16 (102,17.4%)	HPV58 (79,13.5%)	HPV52 (62,10.6%)	HPV18 (62,10.6%)	587	14.7
Xainza	4,600	2377	134	5.6	308	13	91	20.6	HPV16 (69,15.6%)	HPV18 (65,14.7%)	HPV31 (65,14.7%)	HPV51 (45,10.2%)	442	18.6
Baingoin	4,700	1758	68	3.9	174	9.9	54	22.3	HPV16 (24,19.1%)	HPV18 (14,11.2%)	HPV31 (12,9.6%)	HPV58 (11,8.8%)	242	13.8
Nyainrong	4,700	1344	43	3.2	137	10.2	45	25	HPV58 (39,21.7%)	HPV16 (28,15.6%)	HPV31 (17,9.4%)	HPV18 (15,8.3%)	180	13.4
Amdo	5,000	2178	116	5.3	298	13.3	103	25.4	HPV16 (70,17.3%)	HPV31 (68,16.8%)	HPV18(43,10.6%)	HPV58 (40,9.9%)	405	18.6
Nyima	5,100	1727	153	8.9	260	15.1	112	27.1	HPV18 (91,22%)	HPV16 (62,15%)	HPV31 (62,15%)	HPV58 (44,10.7%)	413	23.9
Shuanghu	5,200	428	19	4.4	68	15.9	24	27.6	HPV31 (21,24.1%)	HPV52 (11,12.6%)	HPV18 (10,11.5%)	HPV16 (9,10.3%)	87	20.3

#### 3.1.6 Independent factors influencing HPV infection in Nagqu, Tibet

According to the above findings, it can be mentioned that HPV infection was associated with factors such as women’s age, living locations, and altitude. Our analysis also showed that women with HPV infection had less children and younger age in first delivery. Therefore, we introduced logistic regression to perform multiple-factor analysis to identify the independent factors facilitating HPV infection in Nagqu women. Statistics indicated that age group, living location altitude, and parity were the independent factors influencing HPV infection (**Table 4**). The P-value was 0.031 for those aged between 31 and 35 years and 0.001 for those aged over 50 years. The P-values for altitude and yield were 0.001.

**Table 4:** Independent factors influencing HPV infection in women of Nagqu.

**Factors**		**OR value**	**95% CI**	***p* value**
Age	≤30 years			
	31-50 years	1.315	1.025-1.688	0.031
	>50 years	1.422	1.304-1.551	0.001
Altitude of location	<4,000m			
	4,000-5,000m	2.274	2.044-2.531	0.001
	>5,000m	1.709	1.56-1.872	0.001
Parity	>3			
	≤3	1.212	1.117-1.314	0.001

### 3.2 Cytology

The most common finding was low-grade squamous intraepithelial lesion (LSIL), and 739 women (78.8%) were diagnosed with HPV 16/18. Meanwhile, among other hr-HPV-positive women, only 53.5% (1206 individuals) were diagnosed with LSIL. Additionally, in cytology tests of HPV 16/18 and other hr-HPV-positive patients, the diagnosis of atypical squamous cells of unknown significance (ASCUS), atypical squamous cells cannot exclude HSIL (ASCH), high-grade squamous intraepithelial lesion (HSIL), and cancer was very rare. The most dominant difference was the proportion of Negative for Intra-epithelial Lesions or malignancies (NILM). In the 16/18-positive patients, only 3.1% (29 women) were diagnosed with NILM, whereas in other hr-HPV-positive patients, nearly 40% were diagnosed with NILM. The results are summarized in **Table 5**.

**Table 5:** Cytology and histology results in HPV positives women in Nagqu.

HPV subtypes	HPV16/18			Other hrHPV		
		cases (N)	percentage (%)		cases (N)	percentage (%)
Cytology results	**NILM**	29	3.1	**NILM**	912	39.8
	**ASCUS**	63	6.7	**ASCUS**	84	3.2
	**ASCH**	67	7.2	**ASCH**	59	2.3
	**LSIL**	739	78.8	**LSIL**	1206	53.5
	**HSIL**	36	3.8	**HSIL**	23	1
	**CANCER**	4	0.4	**CANCER**	5	0.2
	**Total**	938	100	**Total**	2289	100
Histology results	**NILM**	174	18.5	**NILM**	435	31.6
	**LSIL**	658	70.2	**LSIL**	801	58.2
	**HSIL**	85	8.9	**HSIL**	132	9.6
	**CANCER**	21	2.3	**CANCER**	9	0.6
	**Total**	938	100	**Total**	1377	100

### 3.3 Histology

A total of 2,315 women underwent histological examinations, as shown in **Table 5**. As shown in **Table 5**, of HPV 16/18-positive patients, 70.2%, 18.5%, 8.9%, and 2.3% of patients had LSIL, NILM, HSIL, and invasive cancers, respectively. In other hr-HPV-positive patients, the diagnosis of NILM was slightly higher (31.6%), LSIL patients were slightly less than HPV 16/18 patients, with a proportion of 58.2%, HSIL was diagnosed in 9.6% of HPV-positive patients, and cervical cancer was found in only 0.6% of HPV-positive women. The histological differences between the two HPV groups were statistically significant (*p* = 0.001).

**Figure 4:**
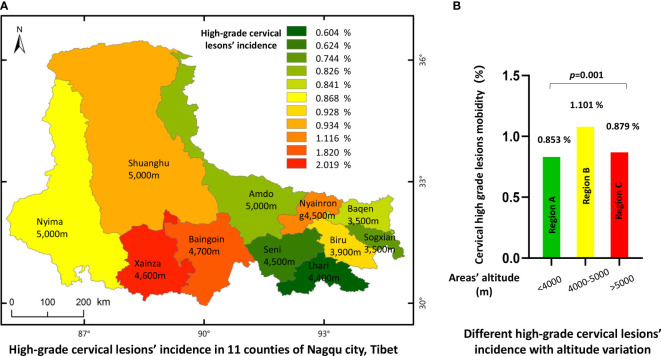
Incidence of high grade cervical lesions in 11 countries of Nagqu. **(A)** High-grade cervical lesions' incidence in 11 counties of Nagqu city, Tibet. **(B)** Different high-grade cervical lesions' incidence with altitude variation.

As there were different HPV infection rates in different counties, we next investigated the distribution of HSIL and cervical cancer lesions in each of the 1 different counties. The analysis indicated that, consistent with the HPV infection profiles, patients with HSIL and cancer lesions seemed to have a similar distribution (**Figure 4A** and **Table 6**). For HPV-positive women, lesions of HSIL and invasive cancer were most prevalent in some high-altitude areas such as Xainza (2.019%), Baingoin (1.820%), and Nyainrong (1.116%), while the incidence was lower in lower-altitude areas such as Sogxian (0.744%) and Baqen (0.826%). After stratifying the lesion rates by the three altitude levels, the HSIL or cancer incidence also presented a significant positive correlation with altitude, as shown in **Figure 4B**, *p* = 0.001.

**Table 6:** Incidence of HSIL or severe lesion among Tibetan women in different areas.

Counties	Number of cases (n)	Percentage (%)
Amdo	18	0.826
Baqen	15	0.841
Baingoin	32	1.82
Biru	40	0.928
Lhari	18	0.605
Nyima	15	0.868
Nyainrong	15	1.116
Seni	25	0.624
Xainza	48	2.019
Shuanghu	4	0.934
Sogxian	17	0.744

## 4 Discussion

To the best of our knowledge, this is the first large-scale population-based survey in Tibet to investigate the prevalence of HPV infection and related cervical lesions in women. This study revealed that the overall HPV infection rate in the women of Nagqu was 13.42%, consistent with recent studies in other high-altitude areas such as Nyingchi, Tibet, 12.81% (20), and Xining, Qinghai, 16.72% (21), while it was slightly higher compared with the investigation conducted about 10 years ago, in which the HPV positivity rate was 9.82% (22). In previous studies, HPV prevalence varied greatly among areas in China and worldwide. Within China, different districts have different HPV infection rates, such as Shanxi (14.8%), Shenzhen (11.3%), Shenyang (17%), Zhejiang (13.3%), and Shandong (18.1% (23–27). According to reports on global levels, HPV-positive rates range from 5% to 10% (28–31). Our data also showed that cervical lesion rates fluctuated significantly among women in different counties of Nagqu Tibet, ranging from 0.605% to 2.019% (**Table 6**).

Intriguingly, there seemed to be a positive correlation between HPV prevalence rate/cervical high-grade lesion incidence and altitude. The higher the altitude, the higher the prevalence of HPV in women. We searched for related studies performed in high-altitude areas all over the world and found that HPV prevalence was high (38.2%) in high-altitude areas of Ecuador, a Latin American country with a largely high altitude, although it was a hospital-based rather than a community-based investigation (32). As mentioned earlier, in China, HPV infection rates at different altitudes in Shanxi were 14.8%, 11.3%, 17%, 13.3%, and 18.1%, respectively. Shanxi, Shenyang, and Shandong are in northern China, whereas Shenzhen, Zhejiang, and Tibet are in the south.

Therefore, we assumed that possible reasons may be attributed to the following two elements: hypoxia from high altitude and cold weather because of extreme climate, both of which facilitate HPV transmission.

Although a few studies have suggested a higher risk of HPV prevalence and cervical cancer in women living in high-altitude areas, there is solid evidence that many types of cancer are more common in high-altitude-living people, such as gastric cancer and breast cancer. The mechanism, which has been comprehensively reviewed (33), is complex and controversial.

Researchers have also revealed an association between cold weather and a high risk of many cancers, such as breast, prostate, and colon cancer. The reasons may be related to evolutionary adaption, high concentrations of certain air pollutants, and low levels of serum vitamin D, although any single factor cannot explain this epidemiological phenomenon (34).

Another possible reason we suspected, although with no scientific data, may be attributed to the social and living styles of Tibetan women, especially in pastoral areas. From our examinations and the subsequent interviews, women in pastoral areas in northern Tibet had very poor sanitation, and many of them did not clean their anogenital areas with tissue or other materials after defecation. In addition, in many places, polyandry and premature sex are common in society. Compared with Han women, Tibetan women have a lower probability of using prenatal care or receiving maternal and child healthcare (35), and Tibetan women have lower knowledge and acceptance of cervical cancer screening (2). All of these behaviors are believed to facilitate HPV transmission.

In our study, we found that the main form of HPV infection was a single infection, while multiple HPV infections were less common, and the results were consistent with those of previous studies. The five most common HPV genotypes in Nagqu people were HPV 16, 58, 31, 18, and 52, accounting for approximately 60% of all infections. This result differed slightly from those of previous studies on the top five HPV genotypes in China (36). Fortunately, all five genotypes were within the nine-valent HPV vaccine spectrum. Thus, there should be an HPV vaccine program initiated in northern Tibet, and our data will provide great value in this effort. The manner of infection in Tibet was approximately the same as that encountered in previous studies in other areas of China (37).

As indicated in other studies, our survey also demonstrated that the age-stratified HPV genotype prevalence has a U-shaped pattern. That is, the infection rate was highest in both the youngest and oldest groups and lowest in the median group (38). This was consistent with previous studies in China that showed a bimodal distribution with age (39). Moreover, the prevalence of HPV genotypes differed according to age group. In the group of women younger than 30 years, the top four HPV genotypes were HPV 16, 31, 58, and 52; in those aged 31–50 years, the top four HPV genotypes were HPV 16, 58, 52, and 18. In women aged >50 years, the top four frequently infected HPV genotypes were HPV 31, 58, 16, and 18. Even when we stratified the women into simple and mixed infection groups, this pattern remained consistent. HPV infection is closely related to women’s age, and women who are sexually active and postmenopausal women have a higher probability of infection with HPV (37, 40, 41), which is also applicable in the Nagqu area. This may be related to factors such as age at first sexual encounter, number of sexual partners, frequency of sexual intercourse, and sexual hygiene for sexually active women (42). The immune capacity of women decreases with age, especially in pre- and postmenopausal women, and the ability to eliminate past and new infections weakens (43). Furthermore, education, economic status, and awareness of cervical cancer and cervical cancer screening are perhaps also the influencing factors (42), although we did not explore this in detail in this research. Therefore, it is necessary to strengthen health knowledge education, improve women’s awareness of self-health care, and conduct regular HPV screening to prevent cervical cancer. Our research may thus be helpful for the prevention of cervical cancer in women in Nagqu.

Although the incidence of HSIL and cervical cancer was nearly the same between the HPV 16/18 infection group and the other hr-HPV infection group, the LSIL frequencies were significantly different (44). In the HPV 16/18 group, LSIL was detected in approximately 70.2% of patients, whereas in the other hr-HPV infection group, LSIL only accounted for 58.2% of women. This phenomenon was also observed in other similar research (45–48). According to our data, HPV16/18 positivity results in different LSIL rates in Tibet compared to non-16/18, which may suggest different management strategies for the two types of infection. Previous studies have shown that persistent HPV infections, such as HPV 16, 18, and 31, are closely related to the occurrence of cervical cancer (49). The occurrence of low-risk HPV is closely related to the occurrence of skin venereal diseases such as condyloma acuminatum (50), suggesting that close attention should be paid to hr-HPV infection in the prevention and treatment of cervical cancer.

As mentioned above, the HPV infection rate correlated with altitude; a similar phenomenon was also observed in the histological results (**Figure 4** and **Table 6**). Some studies have indicated that severe lesions might be attributed to the high prevalence of HPV 16/18 infections instead of the overall HPV infection rate (51), which was also verified in our survey. The top three counties with a high incidence of cervical lesions were Xainza (2.019%), Baingoin (1.820%), and Nyainrong (1.116%), and HPV 16/18 infection rates were also higher in 11 counties (5.6%, 3.8%, and 3.1%, respectively).

The strength of this study is highlighted by the fact that it showed a higher prevalence of HPV infection in women residing at higher altitudes in Tibet. Further analysis indicated that HPV infection and cervical lesion incidence might have a certain correlation with location altitude and it might also vary in different age groups. This information not only provides useful information for the treatment of cervical lesions but also has great value in primary and secondary cervical cancer prevention measures for women residing in high-altitude areas.

The weakness of our survey included missing some important information of the women participating in the study such as the number of sex partner, smoking or alcohol drinking habits, and economic status, so that statistical analyses could not be performed to investigate more risk factors facilitating HPV infection and cervical precancer pathogenesis. Further studies are needed not only for more rigorous and comprehensive investigations but also for long-term surveillance of HPV-positive Tibetan women.

## Data availability statement

The original contributions presented in the study are included in the article/**Supplementary Material**. Further inquiries can be directed to the corresponding authors.

## Ethics statement

The studies involving human participants were reviewed and approved by Ethics Committee of Nagqu people’s Hospital. The patients/participants provided their written informed consent to participate in this study.

## Author contributions

QW, YH, FL, CL, ZS, and DG contributed equally to this work and shared first authorship. DZ, LB, ZH, ZS, NJ, XH, JL, KY, SB, and MT helped in writing this article together. ZX and XX are the authors of this article. All authors have made significant contributions to this article. All authors agree to be responsible for the content of this article and have submitted the manuscript.

## Funding

National Natural Science Foundation of Liaoning, China, 2019-BS-073 and Public health scientific research project of Futian District, Shenzhen hospital of Guangzhou University of Chinese Medicine, NO. FTWS2019104. Interregion collaborative innovation science and technology program, QYXTZX-NQ2022-03.

## Acknowledgments

Science Foundation of Liaoning Provincial Department of Education LZ2019044.

## Conflict of interest

The authors declare that the research was conducted in the absence of any commercial or financial relationships that could be construed as a potential conflict of interest.

## Publisher’s note

All claims expressed in this article are solely those of the authors and do not necessarily represent those of their affiliated organizations, or those of the publisher, the editors and the reviewers. Any product that may be evaluated in this article, or claim that may be made by its manufacturer, is not guaranteed or endorsed by the publisher.
